# Rapid Diagnostic Tests for Identifying Avian Influenza A(H7N9) Virus in Clinical Samples

**DOI:** 10.3201/eid2101.140247

**Published:** 2015-01

**Authors:** Yu Chen, Dayan Wang, Shufa Zheng, Yuelong Shu, Wenxiang Chen, Dawei Cui, Jinming Li, Hongjie Yu, Yu Wang, Lanjuan Li, Hong Shang

**Affiliations:** Zhejiang University, Hangzhou, China (Y. Chen, S. Zheng, D. Cui, L. Li);; Collaborative Innovation Center for Diagnosis and Treatment of Infectious Diseases, Hangzhou (Y. Chen, S. Zheng, D. Cui, L. Li);; National Institute for Viral Disease Control and Prevention, Chinese Center for Disease Control and Prevention, Beijing, China (D. Wang, Y. Shu);; Collaborative Innovation Center for Diagnosis and Treatment of Infectious Diseases, Hangzhou (D. Wang, Y. Shu);; Beijing Hospital Institute of Geriatrics and National Center for Clinical Laboratories, Beijing (W. Chen, J. Li);; Chinese Center for Disease Control and Prevention, Beijing (H. Yu, Y. Wang);; The First Hospital of China Medical University, Shenyang, China (H. Shang)

**Keywords:** avian influenza virus, A(H7N9), rapid diagnostic test, viruses, China, clinical samples, influenza

## Abstract

To determine sensitivity of rapid diagnostic tests for detecting influenza A(H7N9) virus, we compared rapid tests with PCR results and tested different types of clinical samples. Usefulness of seasonal influenza rapid tests for A(H7N9) virus infections is limited because of their low sensitivity for detecting virus in upper respiratory tract specimens.

On March 31, 2013, in China, novel avian influenza A(H7N9) virus infection was diagnosed in 3 persons (*1*). By October 2013, human infection with influenza A(H7N9) virus had reemerged; the number of cases in this second epidemic wave exceeded that of the first wave (before October 2013) (*2*). As of March 10, 2014, the virus had caused 379 human cases and 135 human deaths during both epidemic waves in China (*2*). Because the sensitivity of currently available rapid diagnostic tests (RDTs) for detecting virus in clinical specimens from patients with A(H7N9) virus infection remains largely unknown, we evaluated the sensitivity and specificity of 6 such tests available in China for detecting A(H7N9) virus in different types of clinical specimens from infected patients.

Novel avian influenza A(H7N9) virus has become the most prevalent avian influenza virus strain affecting humans in China. Shortly after the March 2013 outbreak, a real-time reverse transcription PCR (rRT-PCR) for detection of A(H7N9) virus was developed by the Chinese National Influenza Center (*3*). Although rRT-PCR is now considered the standard laboratory-based assay for detecting influenza virus infections, because of its high sensitivity and specificity, it requires high-level laboratory expertise and might not be available in all locations. Thus, the usefulness of RDTs for detecting A(H7N9) virus infection requires assessment. The sensitivity of 6 RDTs has been evaluated in Australia by using a laboratory influenza A(H7N9) virus isolate shared by the Chinese National Influenza Center and the WHO Collaborating Centre for Reference and Research on Influenza in Melbourne, Australia (*4*). However, the suitability of RDTs for detecting A(H7N9) virus in clinical specimens from patients remains largely unknown. We therefore evaluated the sensitivity and specificity of 6 RDTs (Table 1) available in China for detecting A(H7N9) virus in different types of clinical specimens.

**Table 1:** Sensitivity of 6 RDTs for influenza A(H5N7) virus*

RDT	Test time, min	Storage temperature, °C	Shelf life, mo	Detection method	Type of test	Detection limit, TCID_50_/mL†
Wantai Flu A Dot-ELISA‡	20–30	2–8	5	Dot-ELISA	Well, cartridge	10^3^
Wondfo Flu A§	15	4–30	8	Colloidal gold	Well, cartridge	10^3^
Wondfo H7 Subtype¶	15	4–30	8	Colloidal gold	Well, cartridge	10^3^
BinaxNOW Flu A&B#	15	4–30	24	Colloidal gold	Test strip on card	10^4^
ClearView Flu A&B**	15	4–30	24	Colloidal gold	Test strip	10^4^
Kaibili Flu A&B††	15	4–30	18	Colloidal gold	Well, cartridge	ND

*ND, not detected at the highest tested viral concentration (1 × 10^7^ TCID_50_/mL); RDT, rapid diagnostic test; TCID_50_, 50% tissue culture infectious dose. Manufacturer information available at URL for each test.  †Detection limit of A/Anhui/1/2013 (H7N9) virus. ‡http://www.ystwt.cn/flu.html. §http://www.wondfo.com.cn/English/products/List.aspx?MenuID = 050402&ID = 124&temp = 4. ¶Antigen detection kit for human infection with the H7 subtype avian influenza virus, Wondfo Biological Co., Ltd., Guangzhou, China: approved by China Food and Drug Administration after study. #http://www.cdc.gov/mmwr/preview/mmwrhtml/mm6132a4.htm. **http://www.alere.com/ww/en/product-details/clearview-exact-influenza-a-and-b.html. ††http://www.jzmc.cn/content.asp?id=559.

## The Study

The RDTs varied according to detection mechanism, time to results, storage temperature, and shelf life. Of the 6 RDTs, 3 were designed to detect influenza A and B viruses, 2 influenza A virus only, and 1 specifically H7 virus (test names and manufacturer information provided in Table 1). We followed manufacturers’ instructions and visually read the results. At the time of the study, 5 of the 6 tests had been approved for detection of seasonal influenza viruses in China, and approval was still pending for the Wondfo H7 test for A(H7N9). Since then, the Wondfo H7 test has been approved by the China Food and Drug Administration.

To evaluate detection limits of the RDTs, we propagated vaccine candidate A(H7N9) virus strain A/Anhui/1/2013 in MDCK cells and determined the mean 50% tissue culture infectious dose (TCID_50_) per milliliter on the basis of at least 3 independent assays. Viruses were standardized to 1 × 10^7^ TCID_50_/mL and serially diluted 10-fold in phosphate-buffered saline. The detection limit for 3 RDTs was 10^3^ TCID_50_/mL and for 2 RDTs was 10^4^ TCID_50_/mL; 1 RDT could not detect A(H7N9) virus. The following 3 RDTs with the highest sensitivity were chosen for further evaluation of A(H7N9) in clinical specimens: Wantai FluA, Wondfo FluA, and Wondfo H7 (Table 1). The specimens tested were throat swab or sputum (including tracheal aspirates) collected from patients with suspected A(H7N9) virus infection since late March 2013, confirmed by rRT-PCR with primers and probes described previously (*1**,**3*), and stored at −80°C.

To compare the efficiency of RDTs for detecting A(H7N9) virus and seasonal influenza A viruses, we also used RDTs and rRT-PCR to test seasonal influenza A(H3N2)–positive and A(H1N1)pdm09-positive throat swab samples collected during January–April 2012. rRT-PCR testing for seasonal influenza virus was conducted according to the World Health Organization protocol (*5*).

**Figure 1:**
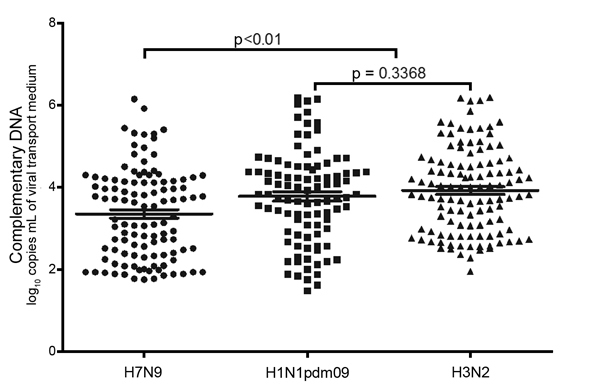
Viral loads of throat swab specimens collected from persons with avian influenza A(H7N9) and seasonal A(H3N2) and A(H1N1)pdm09 virus infection. Statistical analyses were performed by using a 1-way analysis of variance for the 3 groups and an unpaired *t*-test for comparison between the 2 seasonal influenza virus groups. Horizontal lines indicate medians and 95% CIs (above and below means).

In total, 110 throat swab or sputum specimens from 53 A(H7N9)-infected patients and 115 A(H3N2) and 97 A(H1N1)pdm09 throat swab specimens were tested by using the 3 selected RDTs and rRT-PCR; each specimen was prepared and tested by all 4 assays at the same time. As cycle threshold (C_t_) values increased, indicating lower levels of influenza virus in the clinical samples, the sensitivity of RDTs decreased significantly (Table 2). Viral load in throat swab specimens from A(H7N9)-infected patients was significantly lower than that from A(H1N1)pdm09- and A(H3N2)-infected patients (Figure 1). 

**Table 2:** RDT positivity rates for detection of different influenza A virus subtypes in real-time reverse transcription PCR–positive specimens*

C_t_	Wantai Flu A Dot-ELISA†				Wondfo Flu A test, colloidal gold method†				Wondfo H7 Subtype test, colloidal gold method		
H7N9	H1N1 pdm09	H3N2		H7N9	H1N1 pdm09	H3N2		H7N9	H1N1 pdm09	H3N2
<25	7/7 (100)	12/14 (86)	22/24 (92)		7/7 (100)	12/14 (86)	22/24 (92)		7/7 (100)	0/14 (0)	0/24 (0)
25–30	18/38 (47)	35/54 (65)	35/55 (64)		11/38 (29)	23/54 (43)	23/55 (42)		28/38 (74)	0/54 (0)	0/55 (0)
>30	13/65 (20)	8/29 (28)	15/36 (42)		6/65 (9)	7/29 (24)	15/36 (42)		21/65 (32)	0/29 (0)	0/36 (0)
Total	38/110 (35)	55/97 (57)	72/115 (63)		24/110 (22)	42/97 (43)	60/115 (52)		56/110 (51)	0/97 (0)	0/115 (0)

*Values are no. specimens positive by RDT/no. specimens positive by real-time reverse transcription PCR (%). C_t_ , cycle threshold; RDT, rapid diagnostic test. †Sensitivity for influenza A (H7N9) virus was significantly lower than that for either H1N1 pdm09 or influenza A (H3N2) viruses (p<0.01, χ^2^ test), but no statistically significant difference in sensitivity was found between A(H1N1)pdm09 and influenza A (H3N2) viruses.

We then further compared the sensitivity of RDTs for detecting virus in A(H7N9) specimens and seasonal influenza virus specimens with the same influenza A matrix gene C_t_ intervals. We found that for specimens with C_t_ <25, RDT sensitivity for A(H7N9) specimens and seasonal influenza virus specimens was similar. However, for specimens with C_t_ >25, RDT sensitivity was significantly lower when A(H7N9) specimens were compared with seasonal influenza virus specimens with the same C_t_ interval. Overall, RDT sensitivity for detecting A(H7N9) virus was significantly lower than that for detecting A(H1N1)pdm09 or A(H3N2) viruses (p<0.01). Wantai Flu A and Wondfo Flu A detection of A(H1N1)pdm09 and A(H3N2) viruses did not differ significantly (p>0.05). According to the Wondfo H7 subtype colloidal gold kit, 56 (51%) of the 110 A(H7N9) samples were positive and all 212 A(H1N1)pdm09 (n = 97) and A(H3N2) (n = 115) samples were negative (Table 2), demonstrating that this RDT can distinguish between clinical specimens positive for A(H7N9) and seasonal influenza viruses and that its rate of positivity for detecting A(H7N9) viruses is higher than that of the other 2 RDTs tested (Table 2). Ten throat swab samples that were influenza virus negative by rRT-PCR were also negative by the 3 RDTs.

**Figure 2:**
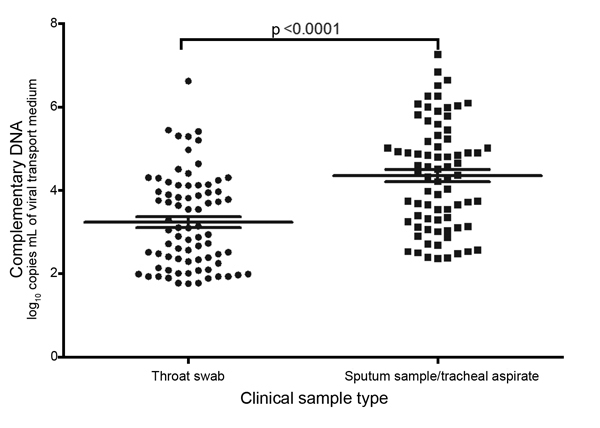
Comparison of viral loads of throat swabs and sputum specimens collected at the same time from persons with influenza A(H7N9) virus infection. Statistical analyses were performed by using a paired *t*-test. Horizontal lines indicate the medians and 95% confidence intervals (above and below means).

Considering that most A(H7N9) virus–infected patients had pneumonia and that the virus replicates more efficiently in the lower respiratory tract than in the upper respiratory tract (*6**,**7*), A(H7N9) viral loads are probably higher in specimens from the lower respiratory tract. Viral loads were significantly higher in sputum/tracheal aspirates than in throat swab samples collected at the same time (Figure 2).

## Conclusions

Although most RDTs examined in this study detected not only seasonal influenza virus but also A(H7N9) virus, the sensitivity of RDTs was lower for A(H7N9) virus than for seasonal influenza virus. Even for specimens with the same C_t_ intervals, RDT sensitivity to A(H7N9) virus was significantly lower than that for either A(H1N1)pdm09 or A(H3N2) virus. The most likely explanation is that cross-reactivity with the nucleocapsid protein–specific antibodies used in RDTs to detect seasonal influenza A virus was significantly lower for A(H7N9) virus. A previous study also indicated that detection sensitivity for swine-origin A(H1N1) viruses varies widely among seasonal influenza A virus RDTs; some tests are unsuitable for detecting several subtypes of avian influenza viruses because of low sensitivity (*8*). 

The Wondfo H7 RDT evaluated in this study was based on a pair of anti-H7 monoclonal antibodies. We found that for each of the 3 C_t_ intervals, the sensitivity for detecting A(H7N9) was relatively higher for the subtype H7 RDT than for the other RDTs.

Our study indicates that throat swab samples, which have been widely used for influenza diagnosis in China, are not suitable for RDT detection of A(H7N9) virus because of the low levels of virus they contain (Figure 1). Viral loads are significantly higher in sputum samples/tracheal aspirates from the lower respiratory tract than from throat swab samples (Figure 2). If any previously designed influenza A virus–specific RDTs are to be used for detection of A(H7N9) viruses, the kits should be modified for use with sputum and tracheal aspirates by improving extraction. In summary, usefulness of currently available seasonal influenza RDTs for diagnosing A(H7N9) virus infections is limited because of their low sensitivity for detecting virus in upper respiratory tract specimens.
